# Supplementary material for: A label-free approach to detect ligand binding to cell surface proteins in real time
Source: eLife. 2018 Apr 26;7:e34944. doi: 10.7554/eLife.34944 (PMC5991833; doi:10.7554/eLife.34944)
Supplement: Figure 1—source data 1. [file elife-34944-fig1-data1.zip › Figure 1 - Source Data 1/00_Info_Figure1-SourceData1.docx]

**Source Data for Figure 1.**

We provide recordings of transient currents as indicated in the table below. Recordings were low-pass filtered at 1 kHz (4-pole Bessel filter) and digitized with a sampling rate of 100 kHz. After data acquisition, the currents were baselined and low-pass filtered at 100 Hz (Gaussian filter).

| **File allocation** |  |
| --- | --- |
| Fig. 1b | Comparison of transient currents induced by either 5-HT (30 μM) or cocaine (100 μM) in paired recordings. |
| Fig. 1d | Current decay rates as a function of cocaine concentration. |
| Fig. 1g | Comparison of transient currents induced by cocaine (100 μM) in presence or absence of extracellular Cl^-^. |
