# Supplementary material for: A label-free approach to detect ligand binding to cell surface proteins in real time
Source: eLife. 2018 Apr 26;7:e34944. doi: 10.7554/eLife.34944 (PMC5991833; doi:10.7554/eLife.34944)
Supplement: Figure 5—source data 1. [file elife-34944-fig5-data1.zip › Fiugre 5 - Source Data 1/00_Info_Figure5-SourceData1.docx]

**Source Data for Figure 5.**

We provide measurements of membrane capacitance as indicated in the table below. For the measurement we used a train of square wave voltage pulses with an amplitude ± 40 mV and a frequency of 200 Hz. The recordings were low-pass filtered at 10kHz (4-pole Bessel filter) and digitized with a sampling rate of 100kHz. After aquisition, the current traces were deconvoluted with the transfer function of the recording device and the passive membrane parameters of a cell were calculated from the theoretical function as described elsewhere (Hotka and Zahradník, 2017). For analysis, data were down-sampled to 50 Hz.

| **File allocation** |  |
| --- | --- |
| Fig. 5d | Apparent ∆C_M_ induced by 100 µM cocaine. |
| Fig. 5f | Concentration-dependent apparent ∆C_M_ induced by cocaine. |
| Fig. 5h | Apparent ∆C_M_ induced by 30 µM 5-HT. |
| Fig. 5i | Concentration-dependent apparent ∆C_M_ induced by 5-HT. |
| Fig. 5j | Comparison of ∆C_M_ induced by either cocaine (100 μM) or 5-HT (30 μM) in paired recordings. |
| Fig. 5l | Apparent ∆C_M_ induced by 30 µM 5-HT in absence of extracellular Cl^-^. |
