# Supplementary material for: A label-free approach to detect ligand binding to cell surface proteins in real time
Source: eLife. 2018 Apr 26;7:e34944. doi: 10.7554/eLife.34944 (PMC5991833; doi:10.7554/eLife.34944)
Supplement: Supplementary file 1. [file elife-34944-supp1.docx]

Figure 4 - supplementary file 1:

Table 1: Model parameters

| **Parameter** | **symbol** | **Unit** | **Value** | **Ref.** |
| --- | --- | --- | --- | --- |
| Outer surface charge density | $\sigma_{o}$ | Cm^-2^ | -0.004 | (Zhang et al., 2001) |
| inner surface charge density | $\sigma_{i}$ | Cm^-2^ | -0.018 | (Zhang et al., 2001) |
| Dielectric constant of a lipid bilayer | $\varepsilon_{b}$ | - | 2.5 ∙ ε_0_ | (Shapiro et al., 2012) |
| Dielectric constant of a bulk solution | $\varepsilon_{Sol}$ | - | 93 ∙ ε_0_ | (Dass, 1986) |
| Dielectric constant of free space | ε_0_ | - | 8.8540 e-12 |  |
| HEK293 cell surface area | $A$ | m^2^ | 2.8274e-09 |  |
| Typical capacitance of a HEK 293 cell | $C_{theoretical}$ | F | 30 e-12 |  |
| Ion valence | *z* | - | ±1 |  |
| Ion concentration | *c* | mM | 150 e-3, 150 e-3 |  |
| Membrane thickness | $\delta_{b}$ | m | 3 e-9 | (Shapiro et al., 2012) |
| Amplitude of a stimulus voltage | $V_{M}$ | mV | 80 |  |
| Faraday constant | *F* | - | 96485 |  |
| Ideal gas constant | *R* | - | 8.314 |  |
| Absolute temperature of a solution | *T* | K | 293 |  |

**References:**

Dass, N. (1986). An International Temperature Dependence of Dielectric Constant in Light and Heavy Water. Phys. Chem. Liq. 15: 323–326.

Shapiro, M.G., Homma, K., Villarreal, S., Richter, C.-P., and Bezanilla, F. (2012). Infrared light excites cells by changing their electrical capacitance. Nat. Commun. 3: 736.

Zhang, P., Keleshian, A.M., and Sachs, F. (2001). Voltage-induced membrane movement. Nature 413: 428–432.
